# Safety and biodistribution of ^111^In-amatuximab in patients with mesothelin expressing cancers using Single Photon Emission Computed Tomography-Computed Tomography (SPECT-CT) imaging

**DOI:** 10.18632/oncotarget.2883

**Published:** 2015-02-04

**Authors:** Liza Lindenberg, Anish Thomas, Stephen Adler, Esther Mena, Karen Kurdziel, Julia Maltzman, Bruce Wallin, Kimberly Hoffman, Ira Pastan, Chang Hum Paik, Peter Choyke, Raffit Hassan

**Affiliations:** ^1^ Molecular Imaging Program, National Cancer Institute, National Institutes of Health, Bethesda, MD; ^2^ Thoracic and GI Oncology Branch, National Cancer Institute, National Institutes of Health, Bethesda, MD; ^3^ Molecular Imaging Program, National Cancer Institute, SAIC-Frederick, Inc, NCI-Frederick, Frederick, MD; ^4^ Department of Radiology and Radiological Science, Johns Hopkins University, Baltimore, MD; ^5^ Morphotek, Exton, PA; ^6^ Laboratory of Molecular Biology, Center for Cancer Research, National Cancer Institute, National Institutes of Health, Bethesda, MD; ^7^ Radiology and Imaging Sciences, NIH Clinical Center, National Institutes of Health, Bethesda, MD

**Keywords:** ^111^In-Amatuximab, radioimmunoconjugates, mesothelioma, pancreatic cancer, mesothelin

## Abstract

Amatuximab is a chimeric high-affinity monoclonal IgG1/k antibody targeting mesothelin that is being developed for treatment of mesothelin-expressing cancers. Considering the ongoing clinical development of amatuximab in these cancers, our objective was to characterize the biodistribution, and dosimetry of ^111^Indium (^111^In) radiolabelled amatuximab in mesothelin-expressing cancers. Between October 2011 and February 2013, six patients including four with malignant mesothelioma and two with pancreatic adenocarcinoma underwent Single Photon Emission Computed Tomography-Computed Tomography (SPECT/CT) imaging following administration of ^111^In amatuximab. SPECT/CT images were obtained at 2–4 hours, 24–48 hours and 96–168 hours after radiotracer injection. In all patients, tumor to background ratios (TBR) consistently met or exceeded an uptake of 1.2 (range 1.2–62.0) which is considered the minimum TBR that can be visualized. TBRs were higher in tumors of patients with mesothelioma than pancreatic adenocarcinoma. ^111^In-amatuximab uptake was noted in both primary tumors and metastatic sites. The radiotracer dose was generally well-tolerated and demonstrated physiologic uptake in the heart, liver, kidneys and spleen. This is the first study to show tumor localization of an anti-mesothelin antibody in humans. Our results show that ^111^In-amatuximab was well tolerated with a favorable dosimetry profile. It localizes to mesothelin expressing cancers with a higher uptake in mesothelioma than pancreatic cancer.

## INTRODUCTION

Mesothelin is a glycosyl-phosphatidylinositol-anchored protein which is normally found in mesothelial cells of the pleura, peritoneum and pericardium [1]. Mesothelin is over-expressed in epithelial malignancies, notably, epithelial mesothelioma and pancreatic ductal adenocarcinoma [2. 3]. Mesothelin binds to CA-125, a specific epitope expressed on MUC16, a transmembrane mucin on cell surfaces of many epithelia. Binding of mesothelin to CA-125 mediates heterotypic adhesion of mesothelin-expressing tumor cells to CA-125-expressing tumor cells and may play a role in tumor metastasis [4]. Recent studies suggest that mesothelin can stimulate pancreatic cancer cell migration via MUC16 mediated signal transduction [5]. The extracellular domain of membrane-bound mesothelin can be shed from tumor cells which when detected in the serum is a useful marker for diagnosis, monitoring and assessment of treatment response in mesothelioma [6]. The differential over-expression of mesothelin in tumors and its role in cell adhesion and tumor metastasis has led to a substantial interest in its therapeutic targeting. Indeed, therapeutic targeting of mesothelin using a variety of strategies including antibodies, vaccines and immunotoxins have yielded promising results and are in various phases of clinical evaluation [7, 8].

Radiolabelling of therapeutic antibodies provides a non-invasive assessment of drug biodistribution and thus could improve dosing strategies and patient selection, enhance understanding of tumor biology and more importantly provide evidence that the antibody actually binds and modulates the intended target. For example, radiolabelling of human epidermal growth factor receptor 2 (HER2)antibody, trastuzumab with Zirconium-89 (^89^Zr) has been used to visualize and quantify HER2-expression in patients with HER2-positive breast cancer [9]. The clearance rate of such antibody radioconjugates in individual patients can also be used to estimate the most appropriate therapeutic dose [10].

Amatuximab (MORAb-009) is a chimeric high-affinity monoclonal IgG1/k antibody targeting mesothelin which is being developed for the treatment of mesothelin-expressing cancers. Amatuximab bound with high affinity to human mesothelin in preclinical studies [11], and was generally well tolerated and showed preliminary evidence of antitumor activity in early-phase clinical studies [12, 13]. Radiolabelled amatuximab could be useful in selecting patients with mesothelin expressing tumors for mesothelin-targeted therapies. In addition, it can serve as a marker for the drug biodistribution and assist in individualizing dosing. In this study, we describe the safety, biodistribution, and dosimetry of ^111^Indium (^111^In)-amatuximab using Single Photon Emission Computed Tomography-Computed Tomography (SPECT-CT) imaging in patients with mesothelin-expressing cancers.

## RESULTS

In this prospective, single institution trial, 7 patients enrolled and 6 patients were imaged. One patient was unable to lie flat on the scanner and therefore was withdrawn from the study. Four patients had malignant mesothelioma and two had pancreatic adenocarcinoma. The median age was 66 (range, 53–73) and included 2 women and 4 men. The actual imaging times averaged over 6 patients for the three scanning sessions were 3.15 +/− 0.67 hours for the first imaging, 26.6 +/− 1.4 hours for the second imaging and 142 +/− 21 hours for the third imaging.

The dosimetry estimates for each patient are shown in Table 1. The effective estimated dose was 0.15 mSv/MBq. The organ which received the highest absorbed dose was liver (0.041 mGy/MBq), followed by the stomach wall (0.023 mGy/MBq.) Excretion of ^111^In-amatuximab was primarily through the hepatobiliary system. Visually, high radiotracer uptake was noted in the heart, aorta, liver spleen and kidneys. The kidneys had an absorbed dose of 0.0015 mGy/MBq. Time activity curves (Figure S1) demonstrated a significant decrease in activity by 24 hours with less than 0.1% of injected dose remaining by 140 hours.

**Table 1:** Mean dosimetry estimates to individual organs

Target Organ	Radiation Dose (mGy/MBq)
Adrenals	9.84E–04
Brain	3.40E–05
Breasts	2.97E–03
LLI Wall	2.12E–02
Small Intestine	4.84E–04
Stomach Wall	2.31E–02
ULI Wall	7.75E–04
Kidneys	1.47E–03
Liver	4.08E–02
Lungs	1.81E–02
Muscle	4.06E–04
Ovaries	1.52E–02
Pancreas	1.71E–03
Red Marrow	8.94E–03
Osteogenic Cells	8.66E–04
Skin	3.23E–04
Spleen	2.08E–03
Testes	6.97E–04
Thymus	1.97E–03
Thyroid	2.41E–03
Urinary Bladder Wall	2.97E–04
Uterus	9.84E–04
Effective Dose (mSv/MBq)	1.45E–01

Table 2 shows the target lesion TBR at the three imaging time points. ^111^In-Amatuximab had the highest TBR at 96–168 hours post-infusion in four patients and at 24 hours in two patients. Patient 1 had a left lower lung target lesion with modest TBRs of 1.2, 2.5 and 3.3 corresponding to the first, second and third imaging time points, respectively. Patient 2 had a left external iliac target lesion with strong focal uptake yielding TBRs of 5, 8.8 and 15.8 at the first, second and third imaging time points, respectively. Patient 3 had a left pleural target lesion with TBRs of 1.4, 9 and 4.6 at the first, second and third imaging time points, respectively. Interestingly, this patient also had a non-target lesion located near the gastro-esophageal junction that demonstrated strong focal uptake and high TBRs of 11.8, 27.5 and 62 at the first, second and third imaging time points, respectively. Patient 4 had a right lower lung target lesion with mild uptake and TBRs of 1.4, 2.2 and 7.6 at the first, second and third imaging time points, respectively. Patient 5 had a pancreatic target lesion with modest TBRs of 2.1, 2.1 and 3.2 at the first, second and third imaging time points, respectively. Finally, patient 7 had a pancreatic target lesion with mild uptake and TBRs of 1.4, 1.5 and 1.5 at the first, second and third imaging time points, respectively.

**Table 2:** Patient demographics and tumor to background ratio of target lesions at the three imaging time-points

Patient	Age/Sex	Diagnosis	Tumor to Background Ratio		
			2–4 hours	24–48 hours	96–168 hours
001	71/F	Mesothelioma	1.2	2.5	3.3
002	53/M	Mesothelioma	5.0	8.8	15.8
003	62/M	Mesothelioma	1.4	9.0	4.6
004	70/M	Mesothelioma	1.4	2.2	7.6
005	54/M	Pancreatic adenocarcinoma	2.1	2.1	3.2
007	73/F	Pancreatic adenocarcinoma	1.4	1.5	1.5

**Figure 1:**
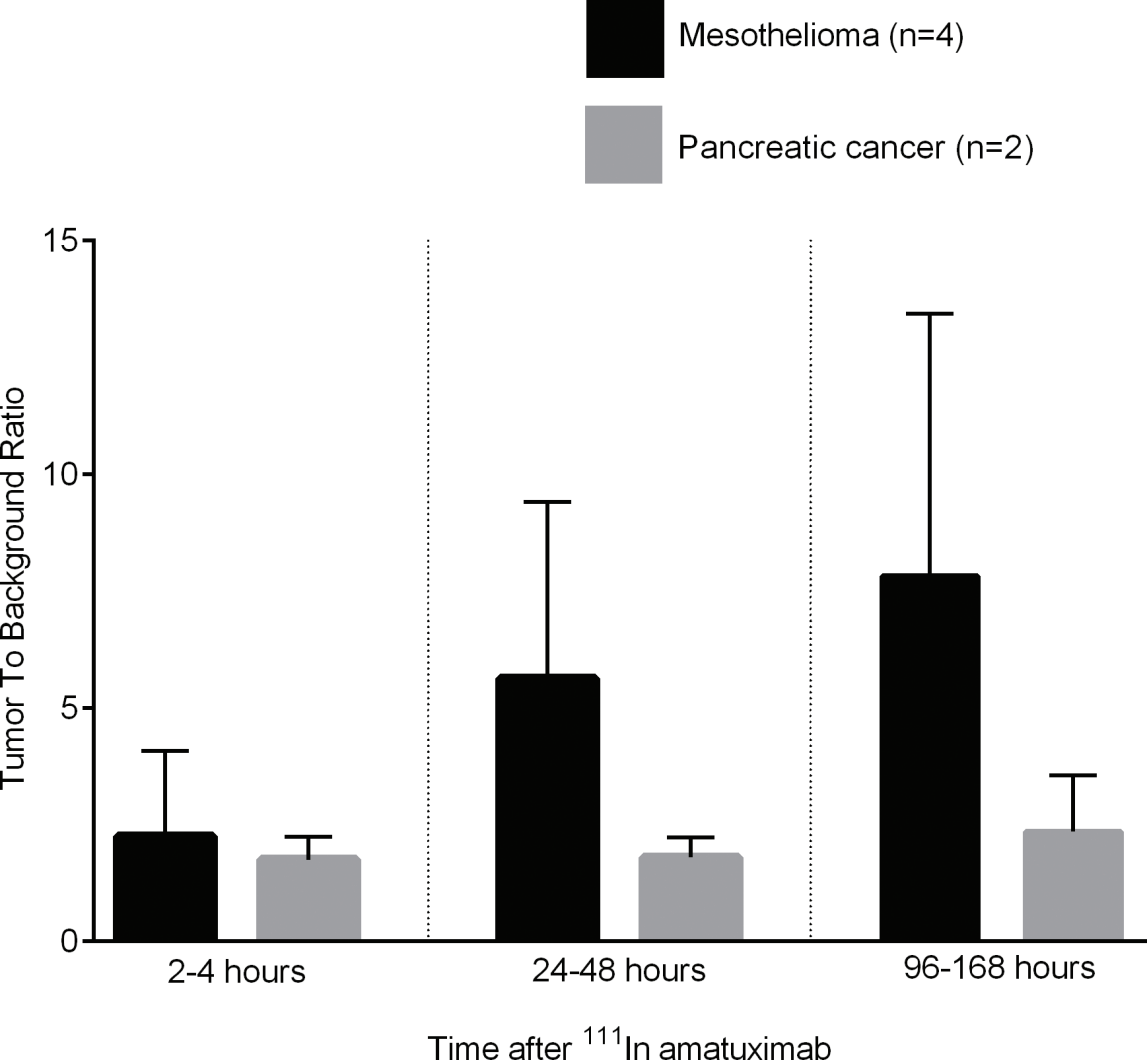
Tumor to background ratios (mean and standard deviation) at 2–4 hours, 24–48 hours and at 96–168 hours after ^111^In amatuximab for patients with mesothelioma and pancreatic adenocarcinoma

For all patients and at all imaging time points, target lesion TBR was at least 1.2 (range 1.2− 62.0), which is considered the minimum TBR that can be visualized.^17^ Figure 1 shows mean TBR at 2–4 hours, 24–48 hours and at 96–168 hours for patients with mesothelioma and pancreatic adenocarcinoma. TBRs were higher for mesothelioma than pancreatic adenocarcinoma at all time points. Relative increases in TBR after ^111^In-amatuximab administration were also higher for mesothelioma. These differences however, were not statistically significant. As expected for monoclonal antibodies, tumor to blood pool ratios were low and ranged between 0.2 to 7.4 (mean 1.5).

**Figure 2:**
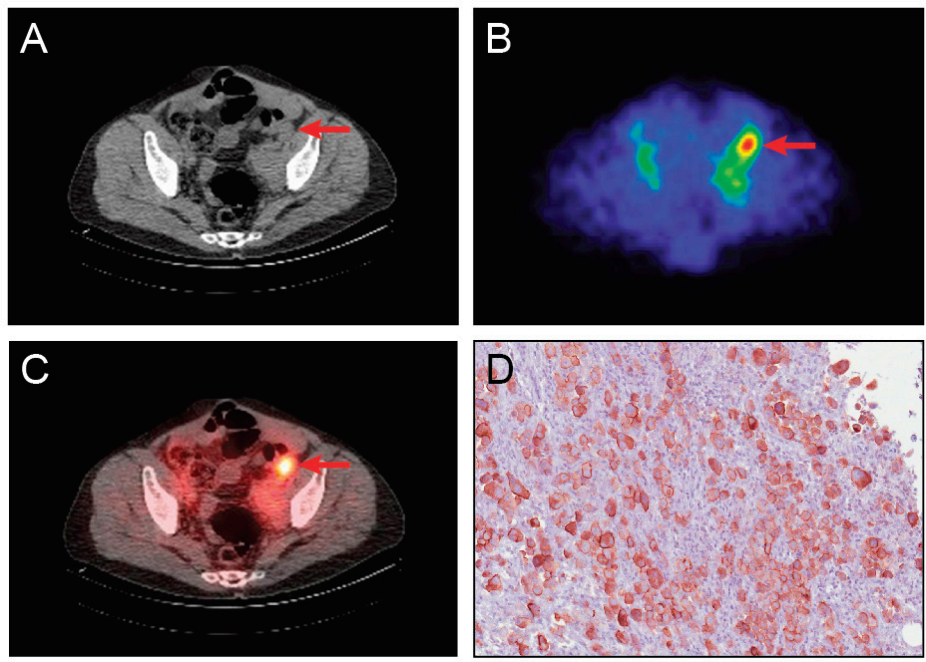
Representative images showing tumor localization of ^111^In amatuximab and tumor expression of mesothelin in a 53 year old man with metastatic mesothelioma CT **(A)**, SPECT **(B)** and SPECT/CT **(C)** fusion image at 24 hours post-injection of ^111^In amatuximab showing focal uptake in the left iliac node (indicated by the red arrow). **(D)** Representative immunohistochemical staining of tumor from left inguinal lymph showing strong membranous and cytoplasmic staining for mesothelin in all tumor cells. Mesothelin staining is indicated by brown staining of tumor cells (20 X magnification).

**Figure 3:**
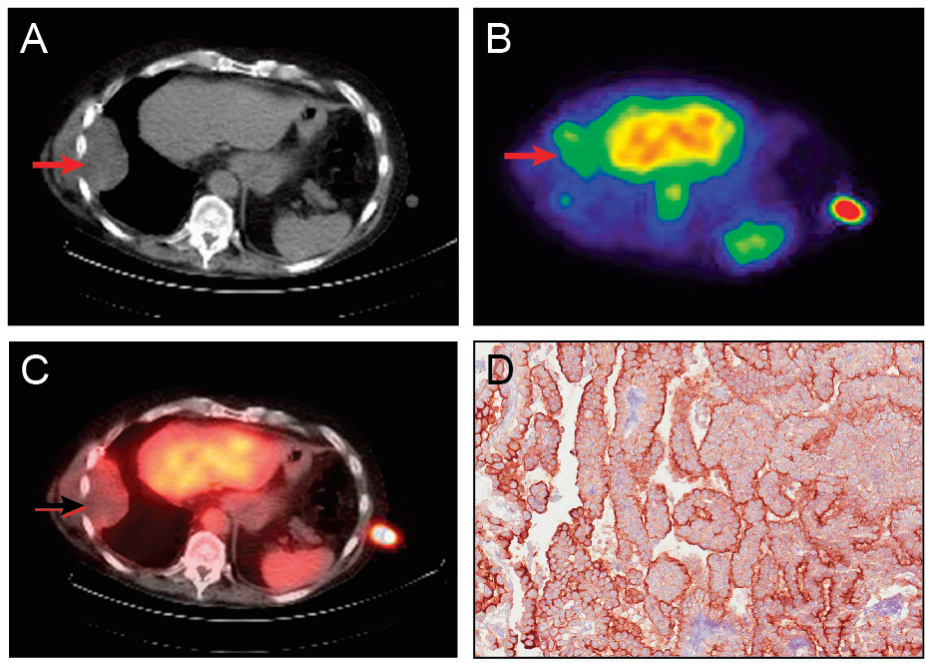
Representative images showing tumor localization of ^111^In amatuximab and tumor expression of mesothelin in a 70 year old man with mesothelioma CT **(A)**, SPECT **(B)** and SPECT/CT **(C)** fusion image at 24 hours post-injection of ^111^In amatuximab showing mild uptake in the right pleural-based mass (arrows). High uptake in the liver, aorta and spleen are physiologic. The increased uptake at the skin level on the left side is the standard vial containing ^111^Indium. **(D)** Representative immunohistochemical staining of tumor from right pleural mass showing strong membranous and cytoplasmic staining for mesothelin in > 90% of tumor cells. Mesothelin staining is indicated by brown staining of tumor cells (20 X magnification).

Representative SPECT/CT images from a 53 year old man with mesothelioma after ^111^In-amatuximab administration is shown in Figure 2A–2D. Intense focal radiotracer uptake is observed in the left iliac node. Tumor from the left iliac lymph node showed strong membranous and cytoplasmic mesothelin expression. Figure 3A–3D shows representative SPECT/CT images from a 70 year old man with mesothelioma after ^111^In-amatuximab administration demonstrating modest radiotracer uptake in the right pleural mass. Strong membranous and cytoplasmic mesothelin expression was seen in more than 90% of tumor cells from the right pleural-based mass.

Two patients had low titers of HACA prior to ^111^In-amatuximab administration. Of the 4 patients who had no detectable HACA at baseline, 3 developed low titers two weeks after ^111^In-amatuximab whereas a fourth patient remained negative for HACA at both the post-treatment time points evaluated. No treatment-emergent adverse effects were observed on this study.

Serum mesothelin and CA125 levels before and after radiotracer injection are shown in Table 3. Serum mesothelin was elevated at baseline in all 4 mesothelioma patients and none of the pancreatic adenocarcinoma patients. Mesothelin levels prior to [mean 5.1 (range 0.6–11.8)] and 14 days after radiotracer injection [mean 7.0 (range, 0–15.4)] were not significantly different (*p* = 0.16). Serum CA125 was elevated at baseline in 4 of 6 patients including both patients with pancreatic adenocarcinoma and 2 of 4 patients with mesothelioma. Compared with pre-injection levels, [mean 303 (range, 5.4–1205)] serum CA125 increased significantly 14 days after radiotracer injection [mean 494 (range, 9.1–1447)] (*p* = 0.03). No association was seen between serum mesothelin or CA125 and tumoral uptake of ^111^In-amatuximab.

**Table 3:** Serum mesothelin and CA125 before and after ^111^In-amatuximab

Patient	Age/Sex	Diagnosis	Mesothelin Levels (nMol/L)*		CA125 (U/mL)^^^	
			Baseline	Day 14	Baseline	Day 14
001	71/F	Mesothelioma	4.4	6.0	86.3	188
002	53/M	Mesothelioma	11.8	15.4	1205	1447
003	62/M	Mesothelioma	3.8	5.8	5.8	9.1
004	70/M	Mesothelioma	9.6	14.8	5.4	15.0
005	54/M	Pancreatic adenocarcinoma	0.6	0.5	52	143
007	73/F	Pancreatic adenocarcinoma	0.6	0	466	1165

* ≥ 1.5 nMol/L is considered abnormally high

^ Normal range is 1.9–16.3 U/ml

## DISCUSSION

This is the first study to show localization of an anti-mesothelin antibody in tumors of patients with mesothelin-expressing cancers. Our results show uptake of ^111^In-amatuximab in tumor sites of patients with mesothelioma and pancreatic adenocarcinoma with higher TBRs for mesothelioma. In addition, we show that the radiotracer was well-tolerated and physiologic uptake was demonstrated in the heart, liver, kidneys and spleen. Favorable dosimetry estimates were obtained in comparison to similar agents in the literature [14, 15]. Radiolabelled amatuximab could be useful in selecting patients for mesothelin-targeted therapies. It could also be used to monitor response to such therapies and serve as a marker for the drug biodistribution and assist in individualizing dosing.

Limited dosimetric information is available on ^111^In-labeled monoclonal antibodies, but in comparison to previous agents studied, ^111^In-amatuximab's dosimety estimates appear favorable [15–16]. Smith-Jones *et al* studied ^111^In-DOTA-MORAb-003, a humanized antibody against folate receptor alpha in three patients with recurrent epithelial ovarian cancer and found average initial uptake of 9% in liver and 0.96% in spleen compared to our findings of 22% in liver and 2% in spleen [15]. Buijs *et al* reported dosimetry for an ^111^In-labelled monoclonal antibody fragment against ovarian cancer with an effective dose equivalent of 0.4 mSv/MBq, which is higher than with ^111^In-amatuximab [16]. Other researchers similarly described higher absorbed dose estimates for target organs such as the liver [17, 18].

Evaluation of the SPECT data using quantitative methods minimized biases inherent to subjective assessments. All measured lesions had calculated uptake at least 1.2 times their respective reference background. The traditional monoclonal antibody, IgG, normally achieves best tumor target concentration in 1–2 days, in part due to its bulky (~150,000 Da) size slowing its kinetics through the vasculature [19]. Circulating antibodies in the blood are still high, so visualization of radiolabelled antibodies takes several more days for free antibody to clear before tumor targeted antibodies can be distinguished from blood pool. Smaller antibody structures such as single chain fragments, rapidly clear the bloodstream, but also have less tumor uptake because of lower avidity and lower overall antigen affinity [20]. This study shows that ^111^In amatuximab has the best TBR mainly at 96–168 hours post-infusion as would be expected for an antibody imaging agent, but at 24 hours also, TBR was visually distinct. Prior to such time points the non-specific background obscured the tumor uptake. Patient 3 had TBR of 9 at 24 hours which decreased to 4.6 at 96–168 hour in a lung mass. This could possibly be explained by weak binding to a small number of mesothelin receptors in the tumor. Patient 7 had low TBRs throughout the imaging period in a pancreatic mass and may not have had adequate binding sites or could be because the antibody tracer was unable to properly engage the receptor.

We observed exceptionally increased uptake in two distant metastatic lesions (left external iliac node in patient 2 and gastroesophageal mass in patient 3) in contrast to uptake within primary tumor sites which were more modest. TBRs at 24 hours were 8.8 in the left external iliac node and 27.5 in the gastroesophageal mass compared to the average of 3.5 in other mesothelioma and pancreatic tumors. It is unclear why secondary lesions might have higher uptake of ^111^In-amatuximab than primary tumors. Despite high specificity of antibodies, nonspecific accumulation of radiolabelled antibodies may possibly occur due to clearance and vascular permeability that does not necessarily indicate antigen distribution [19]. Additionally high interstitial pressure in solid tumors may prevent a sizable percentage of radiolabelled antibodies from interacting with target antigens in the primary tumor [20]. The differential uptake in primary and metastatic sites could possibly be due to their biological differences.

Shed mesothelin is another factor that could interfere with amatuximab binding. Present in the circulation, shed antigen can also be found in the extracellular space [21]. The concentration of shed mesothelin correlates with tumor size and binding of amatuximab to shed antigen decreases available antibody for tumor uptake. Saturating levels of unlabelled antibody administered prior to labeled antibody can improve radiotracer uptake in the target tumor. Tumoral uptake of a radioactive antibody could affect the dose of unlabelled antibody: larger tumors may require larger doses of unlabelled antibody whereas smaller tumors may have higher uptake of the radiolabelled antibody without the requirement for cold antibody [22].

In this cohort of patients, serum biomarker levels were consistent with the published data. Patients with mesothelioma had consistently higher serum mesothelin levels than patients with pancreatic cancers [23]. No significant changes in mesothelin levels were noted after radiotracer injection. The increase in CA125 14 days after ^111^In-amatuximab is consistent with our previous studies in larger cohorts [24]. The increase in serum CA125 after amatuximab (including the dose of cold amatuximab) is likely due to amatuximab inhibiting the binding of CA125 to mesothelin on mesothelial cells [24]. In patient 7, an increase in CA125 was observed even without pre-treatment with cold antibody. It is unclear whether the low doses of amatuximab associated with the labeled conjugate could be responsible or whether this is due to other changes in the tumor.

One of the major limitations of this study was the small number of patients who were assessed. This was an imaging study in patients with advanced cancers with no therapeutic implications. As such we realized that such a study would be hard to accrue and our goal was to provide a proof of concept of target localization of amatuximab in a modest number of patients. Pathologic confirmation of mesothelin expression was not required for study entry for pancreatic adenocarcinoma and mesothelioma considering that these tumors show high mesothelin expression in nearly all cases. Other limitations included variability in the post-injection imaging time-points and the use of organ volume approximation for the dosimety estimates. Notwithstanding the limitations, our results show that a radiolabelled anti-mesothelin monoclonal antibody localizes to the primary and metastatic sites of mesothelin-expressing cancers. These findings will have broad applicability in the clinical development of mesothelin-targeted therapies.

## METHODS

### Patients

Adults with histologically confirmed pancreatic adenocarcinoma, mesothelioma, non small cell lung cancer (NSCLC) or ovarian cancer, with a measurable non-hepatic lesion ≥ 1.5 cm, which had progressed during prior therapy and normal hematologic, hepatic and renal functions, were eligible. Confirmation of mesothelin expression by immunohistochemistry (IHC) was not required for pancreatic adenocarcinoma and mesothelioma which expresses mesothelin in nearly all cases, whereas it was required for ovarian cancer and NSCLC. Exclusion criteria included prior mesothelin targeted therapy, immunomodulatory therapy (e.g. interferon, immunoglobulin therapy, interleukin 1 receptor antagonist) or systemic corticosteroids within 3 months, chemotherapy, biologic therapy, radiation therapy or immunotherapy within 3 weeks, known brain metastases, known prosthetic devices that would cause a radiographic artifact in the target lesion, evidence of other malignancy requiring treatment, clinically significant heart disease, or an electrocardiogram demonstrating clinically significant arrhythmia. The study was approved by the National Cancer Institute Institutional Review Board and the Radiation Safety Committee. All patients provided written informed consent.

### Drug administration

Amatuximab chelated to CHX-A” DTPA was manufactured by Goodwin Biotechnology, Inc., and radiolabelled with ^111^In as previously described [22]. Radiochemical purity was assessed for each dose with ≥ 98% purity by two methods of paper chromatography in addition to internal chromatographic reference. Quality control of the labeled antibody included the LAL (Limulus amoebocyte lysate) endotoxin test and sterility testing. Unlabeled amatuximab was administered in the first five patients at a dose of 50 mg intravenously to saturate any non-specific binding and shed antigen. This dose was estimated based on prior experience with similar radiolabelled antibodies wherein the “cold” dose was needed to improve the tumor to background ratio (TBR). Patients received acetaminophen and diphenhydramine by mouth 30 minutes prior to reduce infusion reactions. Within six hours of cold antibody infusion, approximately 5 mCi of ^111^In-amatuximab was injected intravenously. Unlabelled amatuximab was not administered in the last patient to assess tumor uptake with radiotracer alone. Vital signs and adverse events were monitored after each imaging period as well as at follow-up 2 weeks after radiotracer injection. Thirty days after radiotracer injection an additional follow-up for adverse events was performed by telephone.

### SPECT imaging and image analysis

Following infusion of ^111^In-amatuximab, serial SPECT/CT images were obtained at 2–4 hours, 24–48 hours and 96–168 hours. SPECT/CT imaging consisted of a two bed torso SPECT scan accompanied by a low dose CT scan performed on a Philips Precedence SPECT/CT camera with medium energy high resolution collimators. The images were corrected for uniformity, linearity, energy, center of rotation and attenuation using a CT image derived attenuation map.

Images were evaluated on a workstation running Mim Software version 5.6.7 (Mim Software Inc., Cleveland, OH). Volumes of Interest (VOIs) were manually drawn over target lesions (identified on prior diagnostic anatomic imaging studies) and representative regions of normal organs. Maximum and average activity concentrations were measured. Tumor to regional background ratios were obtained based on maximum count rates. Approximately 1cm VOIs were drawn over gluteal muscle, myocardium, lung, liver, vertebral body, kidney, pancreas, spleen and the right atrium. Using the human standard organ weight and density provided by OLINDA [25], the organ volume was calculated and the activity concentration was converted into the total activity within each of these organs. VOI's encompassing the whole volume of the gut and bladder were obtained, thus providing a direct measure of the total activity within these organs. The VOI over the gut was conservatively drawn to avoid other retroperitoneal structures. From the total activity, either calculated from the average activity concentration or measured directly, the percent injected dose was calculated for each organ.

A 15 ml vial containing 40 uCi at time of injection of ^111^Indium in 10cc of solution was used as a standard and placed in the field of view at every scan. The activity in the vial was measured in the same dose calibrator used to measure the injected dose before each scan. A VOI was drawn around the standard and the total number of counts recorded. The measured activity of the standard and the injected dose was recorded in μCi. From this and the total number of counts measured on the SPECT image, a calibration factor of Counts/μCi was calculated. Using this calibration factor, the total injected dose was converted from μCi into the total number of counts. Therefore, the percent injected dose for each organ is equal to the total counts in the organ divided by the total counts injected. Decay corrections were not performed for dosimetry calculation.

From the activity measurements taken at the three time points, time activity curves were generated (Figure S1). From these curves the residence times were measured by calculating the area under each curve (Table S1). An exponential function was used to estimate the decay curve between the first and second time points and the second and third time points. Finally, the tail of the time activity curve was estimated from fitting a decay curve of ^111^Indium. The time activity curves were normalized to percent injected dose at time of injection. Dosimetry was calculated using OLINDA version 1.1. [25] In the models input form, the male model was selected which is standard for performing dosimetry with OLINDA in humans. In the kinetic input form, the residence times were entered for each organ except for the GI tract organs. Instead, the ICRP 30 GI tract model was selected using a fraction activity of 0.07 entering the gut through the small intestine. No bladder voiding models were used due to the long half life of ^111^In. Background used for TBR was regional depending on the tumor (lung, soft tissue) to provide the most accurate visual assessment.

Overall, the methods used to measure the total activity per organ closely approximates other bio-distribution/dosimetry methods published elsewhere [26], except for a correction applied to the organ weight extrapolated from the subjects total weight. It was deemed this final weight correction unnecessary since the uncertainty in the dosimetry estimate is dominated by the small number of subjects in the study.

### Serum mesothelin, CA-125, tumor mesothelin expression and human antichimeric antibodies

Serum mesothelin and CA-125 levels were measured using the Mesomark assay (Fujirebio Diagnostics, Inc., Malvern, PA) and an automated commercial assay respectively, prior to and 14 days after ^111^In-amatuximab. Tumor mesothelin expression was assessed retrospectively in mesothelioma and pancreatic adenocarcinoma patients with available tissue using monoclonal antibody 5B2 (Novocastra/Leica, Bannockburn, IL) as previously described. [27] Serum human antichimeric antibodies (HACA) to amatuximab were evaluated prior to, 24 to 48 hours after, and 14 days after radiotracer administration using enzyme-linked immunosorbent assay. Serum amatuximab levels were evaluated prior to, 24 to 48 hours after, and 14 days after radiotracer administration.

### Statistical analysis

Serum mesothelin and CA-125 before and after radiotracer administration were compared using the exact Wilcoxon rank sum test. All *p*-values are two-tailed and are presented without any formal adjustment for multiple comparisons.

## SUPPLEMENTARY FIGURE AND TABLE


