# Supplementary material for: Safety and biodistribution of 111In-amatuximab in patients with mesothelin expressing cancers using Single Photon Emission Computed Tomography-Computed Tomography (SPECT-CT) imaging
Source: Oncotarget. 2015 Feb 4;6(6):4496–504. doi: 10.18632/oncotarget.2883 (PMC4414206; doi:10.18632/oncotarget.2883)
Supplement: Supplementary file 1 [file oncotarget-06-4496-s001.pdf]

SUPPLEMENTARY FIGURE AND TABLE

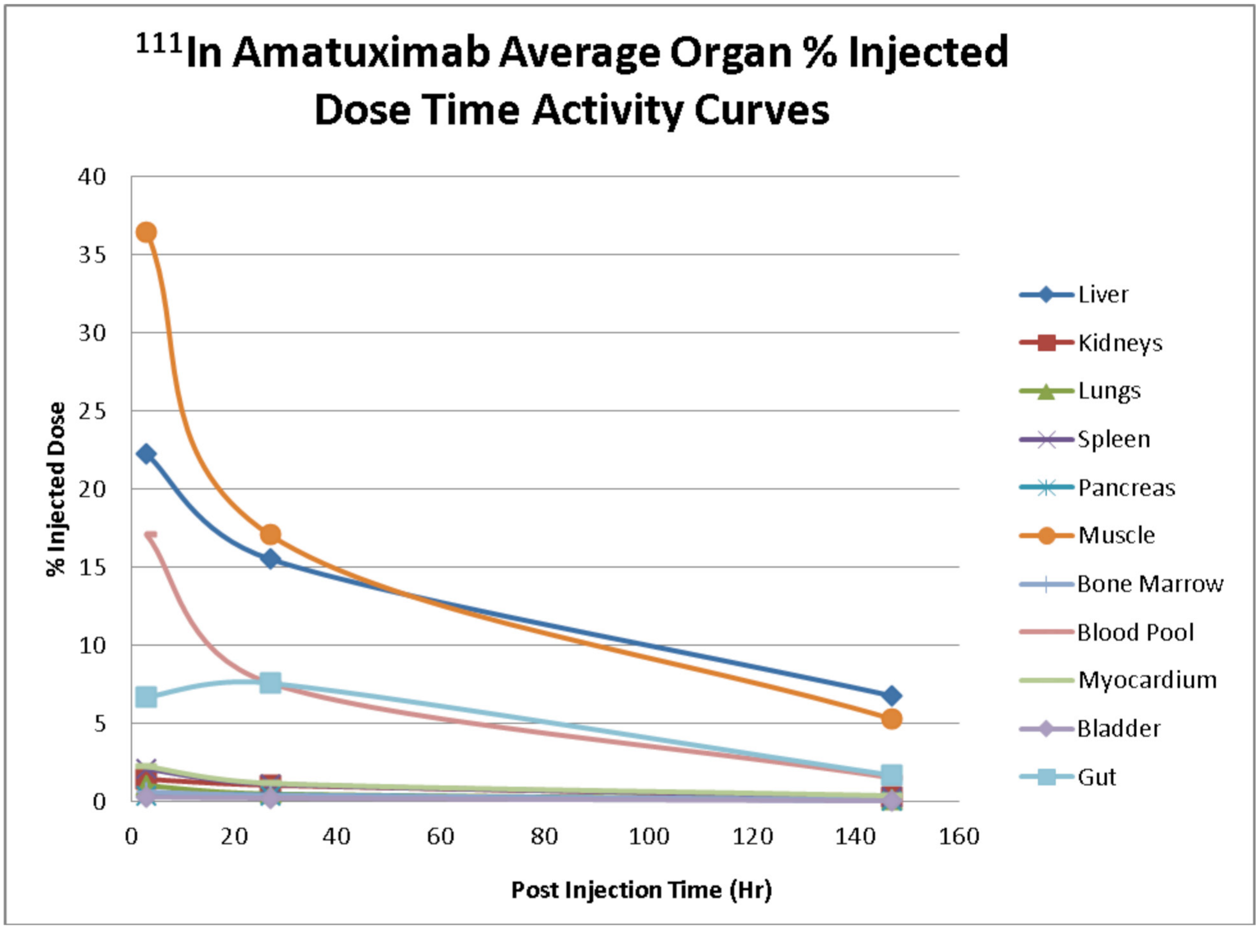

Supplementary Figure S1: % of injected dose- time activity curves averaged over 6 subjects obtained at the three time points. From these curves the radiotracer residence times were determined by calculating the area under each curve. An exponential was used to estimate the decay curve between 3.15 hr and 26.56 hr and 26.56 hr and 142 hr. Finally, the tail of the time activity curve was fitted to a decay curve of ^{111}In. Please see Table S1 which details the residence times measured for each organ.

Supplementary Table S1: Mean residence time estimates for each organ in hours

Organ/Region	Average Residence Time (Hr)
Bladder	0.21 ± 0.05
Gut	7.71 ± 2.33
Muscle	22.16 ± 6.42
Pancreas	0.39 ± 0.11
Blood Pool	8.38 ± 2.45
Bone Marrow	0.48 ± 0.14
Kidney	1.25 ± 0.40
Liver	22.92 ± 6.71
Lung	0.60 ± 0.08
Myocardium	1.47 ± 0.38
Spleen	1.45 ± 0.42
Bladder	0.21 ± 0.05
